# Impact of *Spartina alterniflora* Invasion in Coastal Wetlands of China: Boon or Bane?

**DOI:** 10.3390/biology12081057

**Published:** 2023-07-27

**Authors:** Xiaojun Zheng, Zeeshan Javed, Bing Liu, Shan Zhong, Zheng Cheng, Abdul Rehman, Daolin Du, Jian Li

**Affiliations:** 1 Institute of Environmental Health and Ecological Security, School of the Environment and Safety Engineering, Jiangsu University, Zhenjiang 212013, China; xjzheng@ujs.edu.cn (X.Z.); zeeshan@mail.ustc.edu.cn (Z.J.); ddl@ujs.edu.cn (D.D.); 2 Jiangsu Yangjing Environmental Protection Service Co., Ltd., Lianyungang 222248, China; liubing740219@163.com; 3 School of Energy and Power Engineering, Jiangsu University, Zhenjiang 212013, China; 4 Jiangsu Xianghe Agricultural Development Co., Ltd., Lianyungang 222000, China; aucz000@163.com; 5 School of Earth and Space Science, University of Science and Technology of China, Hefei 230026, China; abdulrehman008@mail.ustc.edu.cn

**Keywords:** *Spartina alterniflora*, coastal wetlands, China, carbon sequestration, biodiversity

## Abstract

**Simple Summary:**

The objective of this comprehensive review is to address some important aspects linked to the spread of *Spartina alterniflora* in China’s coastal wetlands. Firstly, we summarize the mechanisms behind its spread and its ability to adapt to different niches, highlighting its characteristics and strong adaptability. Next, we examine the ecological effects of *Spartina alterniflora* on various aspects of ecosystem function, including habitat conversion, biodiversity alteration, soil carbon flux and sequestration. The presence of *Spartina alterniflora* in China has significantly changed the structure and configuration of coastal wetlands. To efficiently manage, increase, or eradicate *Spartina alterniflora*, it is vital to implement a complete and adaptive plan that reflects the long-term suggestions and precise circumstances within different provinces of China.

**Abstract:**

Invasive plants, like *Spartina alterniflora* (SA), have a competitive advantage over native flora due to their rapid utilization of vital soil nutrients. This results in the depletion of resources for native plant species, significantly impacting ecosystem diversity and stability. This comprehensive review addresses several key aspects related to SA’s spread and spatial distribution in China’s wetlands. The rapid expansion of *Spartina alterniflora* is attributed to its high reproductive ability, adaptability to environmental factors like elevated salinity, and ability to disperse its seeds via tides. *Spartina alterniflora* mainly were found in Zhejiang, Jiangsu, Fujian, and Shanghai provinces, accounting for more than 90% of China’s total *Spartina alterniflora* area. *Spartina alterniflora* rapid growth results in displacement of native species and loss of vital microbial, plant, and animal diversity. Some studies reported that *Spartina alterniflora* increases carbon storage, while others argue that it weakens this function. The impact of *Spartina alterniflora* on organic and inorganic carbon requires further research for better understanding dynamics of carbon in coastal wetlands. The controlled growth of *Spartina alterniflora* can be beneficial in many aspects of the coastal wetlands’ ecosystem. In China, various methods have been employed to control the invasion of SA. Physical control, such as removing the plants and converting them into fertilizer or bioenergy, has been commonly used but has limitations like air pollution and the potential for re-invasion. Chemical herbicides like Imazapyr and Haloxyfop-R-methyl have effectively controlled and prevented re-invasion in specific areas, but their potential adverse impacts are still uncertain. Wetland Park construction, aquaculture development, and substituting native or exotic species with mangroves or reed communities have also been successful. It becomes evident that a long-standing and Contextual approach is necessary to effectively manage the advantages and curtail the drawbacks associated with *S. alterniflora* across China.

## 1. Introduction

Coastal wetlands are situated in marine terrestrial interweaved zones, crucial for preserving biodiversity, protecting water resources, and enhancing animal and plant life [1,2]. However, these wetlands are also highly vulnerable to the effects of global change [3]. Coastal ecosystems are referred to areas adjacent to the sea, where sea tides help to create attributed features of typical mangroves and intertidal saltmarshes, which can be vulnerable to the invasions of organisms from outside of their native dispersal range [4,5,6]. These invasions alter the receiving coastal wetlands’ species composition and ecological dynamics [7].

*Spartina alterniflora* (SA) is a grass of C4 type plant categories, immensely found in the Atlantic coastal zones of the World [8]. In December 1979, it was brought to China’s wetlands from the United States to prevent soil erosion and shield the coastline [9]. The initial purpose of bringing this grass specie into China was to use it in ecological engineering for wave protection and embankment reinforcement [10,11,12]. Since then, it has spread across 340 km^2^ of coastal wetlands in China. The invasion of SA is one of the most significant ecological issues in the coastal wetlands of China. It covers 92.4% of the total area in tropical and subtropical coastal wetlands [13].

The extensive expansion of SA vast along the coastal regions of China is because of fast adaptive favorable environmental conditions and hence its fast dispersal capability [14,15,16,17]. The expansion of SA has resulted in changes to various features of wetlands in China, such as soil moisture content, productivity, hydrokinetics, sedimentation, pedogenesis, and nutrient accumulation [18,19]. For instance, SA has become dominant in various parts of China by replacing native plant species [20]. However, the consequences of these alterations differ depending on the location and habitat. The introduced invasive species have positive and negative impacts on the coastal wetlands [21]. Therefore, considering both invasive species’ positive and negative consequences is vital [22]. Studies suggest conflicting results regarding the impact of SA invasion on the carbon sink function of coastal wetlands. While some studies indicate that the invasion of SA enhances the carbon sink function of coastal wetlands [23], others suggest that it weakens this function [24]. The positive impacts include erosion control [25] and nutrient cycling [26]. In some regions, SA contributes positively to protecting the shoreline; in others, it has instigated harm to maricultural processes. At the same time, the negative impacts include habitat alteration [27] and salt marsh loss [28]. SA can influence and enhance heavy metal concentration in wetlands [29]. SA can also assimilate high phosphorus, resulting in a drastic drop in N:P ration, which could have long-term consequences on nutrient cycling mechanisms. This nutrient imbalance may decrease the coastal ecosystem’s reproductive capability [24]. According to Zhou et al. [30], the presence of SA in Jiangsu Province has led to a significant decrease in macrobenthic diversity.

Furthermore, SA has also caused the loss of 80% habitat of migratory birds and endangered species communities in Yancheng National Nature Reserve (YNNR). The effects of changes related to SA vary depending on the location and condition of the habitat. Previous studies have discussed the advantages and disadvantages of SA and its population dynamics, management strategies, and vast influential diversity.

This review aims to provide an overview of the current research progress on SA in China and find potential research gaps. The present review will be organized as follows: (a) a comprehensive analysis of the spread of SA in China; (b) an evaluation of the influence of this plant on habitat; (c) an assessment of the effects of *Spartina alterniflora* on biodiversity; (d) an examination of the impact of this invasive species on carbon sequestration and (e) a discussion of strategies that can be employed for managing the invasion of *Spartina alterniflora* in China, as well as the systematic supervision of this species to promote sustainable development of coastal regions.

## 2. *Spartina alterniflora* Characteristics and Invasive Potential

*Spartina alterniflora* is characterized by its rigid, upright stems with a maximum of 11 nodes. These stems can grow over 200 cm tall and have a diameter of 1.2 cm. The leaves of *Spartina alterniflora* are approximately 50 cm long and 2 cm wide [31,32,33]. *Spartina alterniflora* root depth characteristically ranges from 8 to 20 cm [34]. The typical flowering period of *Spartina alterniflora* is from August through September [35]. The intertidal brackish plant specie *Spartina alterniflora* grows well in areas with salinity ranging from 8 to 33%, which promotes its growth and reproductive success. Although it can establish itself in freshwater environments, *Spartina alterniflora* appears to be incapable of producing viable seeds under such conditions. Therefore, its ability to thrive and reproduce effectively is closely associated with the ideal salinity levels mentioned above.

*Spartina alterniflora* has a competitive edge over native species in China due to its rapid range expansion and high reproduction rate [32,36,37]. This success is attributed to its high reproductive ability, adaptability to environmental factors like elevated salinity and inundation, and the ability to disperse its seeds via tides, facilitating further establishment in new areas [32,37]. Sexual reproduction through seeds and asexual propagation through tilling are considered the two primary methods for the rapid spread of SA [38]. However, there is a disagreement regarding the comparative significance of sexual and asexual reproduction. According to Xiao et al. [39], the seedlings in spring play a crucial role in the colonization of new habitats and achieving a fast rate of range expansion for *S. alterniflora*. In contrast, some experts believe that asexual reproduction contributes more to the invasive success of *S. alterniflora* along the coast [36].

Several factors contribute to the invasion mechanism of SA in China, including both biotic and abiotic factors [32]. Biotic factors are considered critical for its success, particularly its reproduction ability.

In addition to its biotic characteristics, the purposeful introduction of SA is also an important abiotic factor linked to its invasion success. Table 1 lists biotic and abiotic factors contributing to the invasion of SA in China.

*Spartina alterniflora* habitats are in a wide ecological niche, and unlike the majority of species, it is capable of sustaining in a rather challenging saltwater environment. “Niche” is defined as the position or role a specific organism plays within its ecosystem, including resource utilization and environmental interactions. An organism’s niche is determined not only by the biotic characteristics but also by the abiotic factors of the surroundings. However, the role of biotic factors in shaping an organism’s niche is significant, as these factors greatly influence an organism’s interactions, adaptability, and resource availability in the ecosystem. Biomass is directly proportional to the richness with higher biomass associated with greater abundance of the particular species and hence considered a biotic factor. In the northern coastal wetlands of China, *Suaeda* is the dominant native intertidal vegetation, but compared to *S. alterniflora*, this species has a smaller biomass. Among the abiotic factors, intentional introduction is the major factor contributing to invasion. It can be inferred that had the deliberate introduction not happened, the invasion would not have occurred. *S. alterniflora* has a high reproduction rate hence spreading quickly. Rapid reproduction rates and a higher range of expansion give this species a competitive advantage over native species, thereby facilitating invasion.

## 3. Spatiotemporal Distribution of *Spartina alterniflora* in China

**Figure 1:**
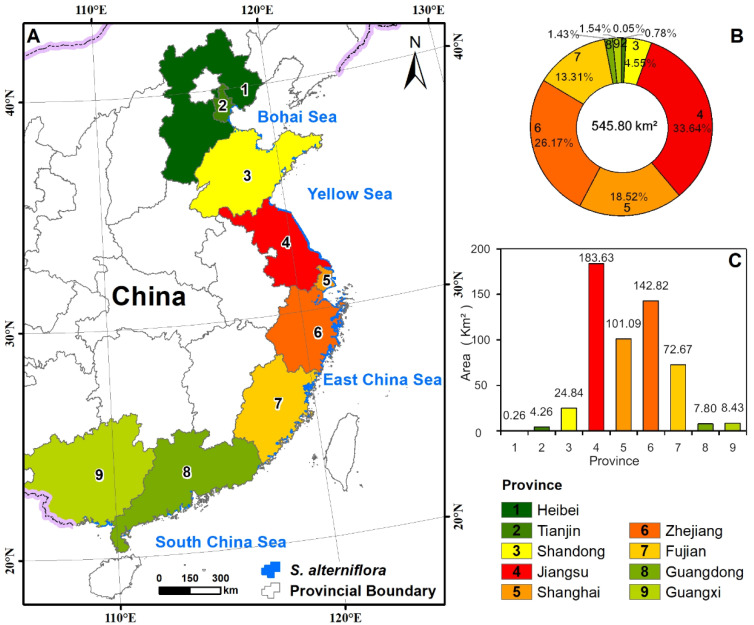
*Spartina alterniflora* spatial distribution in coastal wetlands of China, (**A**) Spatial distribution, (**B**) areal proportion, and (**C**) total area. Adopted by [14].

The control and invasion of exotic species have become a prominent topic in the eco-environmental community worldwide. Governmental agencies, scientific communities, and citizens must have access to information on salt marshes’ spatial distribution, species composition, and areal extent over an extended period. Despite national and international concerns, there is a dearth of publicly available data on salt marshes in China at a national scale. *Spartina alterniflora* has been extensively classified and mapped using satellite remote sensing technology in recent years. Several researchers have utilized freely available satellite images, including Landsat images, for this purpose, as demonstrated in studies conducted by Mao et al. [40], Wang et al. [41], and Zeng et al. [42]. Figure 1 shows a spatial spread of SA in China.

The distribution of SA is unevenly spread across various provinces in temperate and sub-tropical coastal China. The provinces with the largest areas of *Spartina alterniflora* were Zhejiang, Jiangsu, Fujian, and Shanghai, which accounted for more than 90% of the total *Spartina alterniflora* area in China. Over time, the expansion process is divided into three stages: the growth period, the outbreak period, and the plateau period. From 1990 to 2018, *Spartina alterniflora* in Zhejiang, Jiangsu, and Fujian experienced two phases of rapid increase, one from 1990 to 2000 and the other from 2010 to 2018, while a slightly increasing phase between 2000 and 2010. However, in Shanghai, *Spartina alterniflora* increased rapidly after 2005 [14,40,42,43].

1. Effects of the invasion of *Spartina alterniflora* invasion on habitat

Significant alterations in wetland habitats across China have been caused by *Spartina alterniflora* by competitive exclusion [11,44,45]. For instance, pioneer species at Chongming Island, like *Phragmites communis* and *Scirpus mariqueter*, were negatively impacted [46]. Radically altered habitats are created by invading this species with elevations from formerly non-vegetated marshes to vegetated populations in areas with void vegetation and zero competition [47]. Although the expansion is favorable and highly productive for coastal animals, it tends to threaten the local ecosystem with narrow intertidal zones [48]. SA salt marshes are considered to be one of the most productive ecosystems in their native range, with rich food sources for benthic communities as well as a habitat for waterfowl and forage [11]. However, its introduction to China as an exotic and invasive species results in a starkly dissimilar community structure and function compared to its native habitat. Nutrient structure, biodiversity of the benthic community, and metrics of several biological indicators have been severely affected by the invasion of this species [49]. Nonetheless, the production and habitat for SA in China are currently in the initial stages. Formulating a stable ecosystem may take a while, and the potential for enhanced production might require a long time.

SA is known to thrive in the upper region of tidal estuarine beaches along the coast of, for instance, Jiangsu province, where plants have previously been unoccupied [50]. Due to the lack of competition, SA can quickly establish itself in these areas [51]. This growth of SA into previously unvegetated regions can provide additional productive habitation for tidal species. Nevertheless, if the littoral zone is slender, this growth can significantly threaten the ecosystem.

2. Impacts of *Spartina alterniflora* invasion on biodiversity

Owing to its invasive nature, this species can have various impacts on biodiversity in China depending on the type, age, and location of the habitat being invaded. Strong adaptability and resilient biotic characteristics make this species grow and spread rapidly while out-competing native flora, causing coastal wetlands’ loss of crucial biodiversity (microbial, plant, and animal diversity) [52].

Studies have shown that the invasion of SA in China’s coastal zones has resulted in the displacement of salt marsh plants, encroachment into gaps of mangroves, and the transformation of mudflats into salt marshes [53,54]. Over the past few decades, SA has been widely reported to invade native ecosystems such as *Phragmites australis*, *Suaeda salsa*, Sea syringum, and mangroves, and it is now becoming the dominant vegetation in most coastal areas of China. Coastal wetlands are ecosystems at the boundary between land and sea, where soil salinity variations can span from low to high levels, impacting plant competition. SA exhibits greater salt stress tolerance than the indigenous *P. australis* species and has out-competed native plants *P. australis* and spread to unsuitable areas [55]. *P. australis*, with its restricted tolerance for salinity, exclusively thrives in habitats characterized by low levels of salinity. SA and *P. australis* competition changes along the salinity gradient [56]. As salt stress increases, the growth of native *P. australis* is driven more by stress than competition, whereas competition remains vital for growing SA. Zhang et al. [54] reported that SA demonstrated a gradual displacement of mangroves within the intermediate salinity areas of southern Chinese estuaries.

Benthic fauna is critical as a trophic hub in the coastal wetland food web. Still, they are highly vulnerable to external disturbances, which can affect the entire wetland ecosystem. One of the serious threats facing coastal wetlands in China is the invasion of SA, causing a decline in the density of bivalve mollusks and nematode trophic diversity [31]. While the invasion of vegetation-free flats initially increases the diversity of benthic fauna due to increased food sources, the diversity of community structure decreases as the invasion time prolongs. The diversity of community structure decreases, and the source of food organic carbon will change to a single one, which will have a certain impact on the food web’s energy supply diversity and structural stability. The open light flat in the coastal wetland intertidal zone is an important part of the salt marsh and mangrove ecosystem and the main foraging ground for birds. It is also more likely to be occupied by the expansion of SA, which seriously threatens the survival of bivalves and other species suitable for light beach habitat [32], and further affects the high nutrition. Hierarchical animals reduce the food source of birds and some fish, which is not conducive to the survival and migration of birds and needs to be paid attention to in management and protection [57]. Invasion time has been reported to be an important factor determining the impact of SA on benthic fauna. Although much research has been carried out on the impact of SA on benthos communities, there are still deficiencies, and several aspects need to be strengthened.

Research has shown that SA invasion changed the soil microbial community structure in the mangrove ecosystem, particularly the bacterial community [58]. Soil bacteria play an important role in biogeochemical cycles, including carbon, nitrogen, and sulfur, in wetland ecosystems, and microorganisms are critical in cases of plant invasion [59]. The growth of SA has been found to raise soil sulfur content in coastal wetlands, which can affect the soil microbial community [60]. The invasion of SA led to a significant decrease in the relative abundance of Bacteroidetes in sediments, becoming a non-dominant genus, and a significant increase in the relative abundance of Proteobacteria iron-reducing bacteria [61]; at the same time, the dominant genera of soil iron-reducing bacteria were succeeded. After the invasion of SA, the abundance of the dominant genus Desulfuromonas in the wetland was significantly reduced and became a non-dominant genus, while anaerobic myxobacteria (Anaeromyxobacter) increased significantly and became the third dominant genus. However, one study found that the invasion of SA enhanced fungal diversity was observed, leading to modifications in the structure of the fungal community. This, in turn, facilitated the proliferation of saprotrophic and pathogenic fungi while diminishing the impact of random processes in the fungal community assembly [62]. The effects of controlling SA on soil microbial communities remain poorly understood, and more research is needed to better understand the impact of SA on microbial biodiversity.

3. Impacts of *Spartina alterniflora* on Carbon sequestration

Coastal wetlands are crucial in mitigating global climate change and serving as habitats for diverse organisms due to their assimilation of atmospheric carbon and provision of various ecosystem services [63]. Although it covers only 4–6% of the World’s land area, coastal wetlands accounts for 20–30% of the World’s carbon sequestration capacity [24]. However, these wetlands are rapidly degrading due to human activities such as urban expansion and land reclamation for commercial and recreational purposes. The impact of SA invasion on the carbon sink function of these ecosystems and the underlying mechanisms are still unclear. While some studies suggest that the invasion of Spartina alterniflora enhances the carbon sink function of coastal wetlands, others argue that it weakens this function.

SA has a high photosynthetic rate, primary productivity, and biomass, promoting organic matter input into sediments. This leads to changes in the ratio of carbon to nitrogen (C:N) and carbon to sulfur (C:S) and creates different soil microbial environments. The root exudates of SA also change physicochemical properties such as soil pH and Eh, resulting in a higher soil organic carbon (SOC) retention capacity compared to local plants such as *Suaeda salsa*. Studies have shown that total sulfur levels and soil organic carbon in the sedimentary systems invaded by SA were significantly higher than those in adjacent local sedimentary systems, and carbon storage increased accordingly [64,65]. The Soil carbon storage increased with the extension of invasion time [65,66]. The higher C:N and lignin content in SA can be attributed to the slower degradation rate of its litter. Research has shown that the degradation rate of SA litter is 59.79% slower than native plants. This slower degradation rate results in organic matter accumulation in the sediments, leading to increased carbon storage [67]. Therefore, the slower degradation rate of SA’s litter is an important factor in the increased carbon storage of sediments. Carbon sequestration has been observed to be increased by 18.96% to 40.24% and 4.66% to 32.04% from 0 to 20 cm and 20 to 40 cm layer, respectively, by SA, mainly due to the high stability of soil structure on coastal wetlands, across the chrono-sequence from 4 to 12 years [68]. Another earlier study showed increased carbon sequestration from 27% to 69.6% in invasion time 8 to 14 years of SA in the upper 0–10 cm layer of soil [69]. The estimated calculations of photosynthetic reaction, SA has an annual carbon sequestration capacity of 2274 g m^−2^ yr^−1^, which is 4.6 times greater than the average for other Chinese vegetation (494 g m^−2^ yr^−1^) [70]. The SOC concentration from different salt marshes invaded by *Spartina alterniflora* reported in different studies is shown in Table 2.

However, some studies indicate that the invasion of SA negatively impacts carbon storage and emissions. For instance, the replacement of native plants with SA has been found to have little effect on soil properties, biomass accumulation, or plant-soil carbon storage. Additionally, while SA invasion may lead to higher sediment carbon storage, higher carbon-containing greenhouse gas emissions offset this. Even the total carbon (TC) content of native plant surface sediments is higher than that of SA swamp, and its invasion may lead to a dramatic drop in soil nutrients, especially carbon content [24,74].

Regarding carbon emissions, the invasion of SA has been found to increase methane emissions due to providing more substrates for soil microorganisms, including the abundance of methanogens [75,76]. Moreover, some studies have found that the short-term increase in the soil carbon content of light flats invaded by SA is caused by the increase in soil organic carbon, while the invasion of SA does not change the soil inorganic carbon (SIC) content. SA in wetlands has decreased SIC reserves, largely due to root transformation and variations in growth characteristics over time [77]. The role of SIC in C sequestration is vital, particularly in coastal saline-alkaline regions. However, previous studies have not thoroughly examined the impact of invasive plant species on soil inorganic carbon in coastal wetlands. This topic has been somewhat explored, but little research has been conducted [27,78,79].

Hong et al. [27] found that SIC is higher in native mudflats as compared to SA marshes. The rise in SIC after the change of *Spartina marshes* to aquaculture ponds can be attributed to managing protocols. For instance, using NH4HCO3 as a fertilizer could promote carbonate precipitation, as seen when coastal marshes transform into croplands [27,79].

The research on the regulation mechanism of SA to the “carbon sink” function of coastal wetlands is still in the early stage and unclear. Therefore, due to the limitations of the research area, time span, research purposes, population differences in invaded areas, and the number of studies, the question of how ecosystem functions, such as carbon dynamics, respond to plant invasion is still unclear. At large scales, how do factors such as topography, climate, and land use patterns affect the spatial heterogeneity of carbon stocks; on a small scale, how do soil texture, nutrient composition, and vegetation heterogeneity before and after invasion (such as reed, *Suaeda salsa*, etc.) affect the distribution of carbon forms. With the intensification of SA invasion, the uncertainty of the dynamics of this huge carbon pool is becoming highly significant, and it is urgent to elucidate its impact process and mechanism from the perspective of a complete ecosystem.

## 4. Control Measures

Physical control, biological substitution, chemical herbicides, or integrated measures were adopted across China to control the invasion of SA [80]. The effectiveness of these control measures depicted spatial heterogeneity across mainland China’s coastlines, with significant success in East China. Physical control involves removing the plants and converting them to fertilizer or bioenergy. It is a commonly employed control measure in China, but it has certain drawbacks, as it causes air pollution and does not prevent succeeding re-invasion [81]. While chemical methods like Imazapyr and Haloxyfop-R-methyl herbicides were proven effective in effectively controlling and preventing re-invasion in Chongming, Shanghai, their potential adverse impacts remain inconclusive and vague [82]. Construction of wetland parks and aquaculture development are some other methods successfully employed to address the invasion of SA.

Additionally, the substitution of native or exotic species with mangroves, the construction of seawalls in salt marshes, mangroves, and the conversion of SA communities to reed communities have also been employed to address the problem [83]. Reduction in alterniflora biomass, enhanced regional habitat, flourishing biodiversity, enrichment in soil nutrition, and improvement in physicochemical properties of sediments were observed by using these methods. In East China’s coastal ecosystems, cognizant reduction of SA positively influenced the density and diversity of native flora and fauna [25].

Generally, once an exotic species has taken hold in a large geographical area, it becomes challenging to eliminate it. In the case of SA, thinking about the forthcoming course of China’s coastal ecosystems over extended temporal and spatial ranges is necessary.

## 5. Conclusions

SA, a perennial grass commonly found in salt marsh environments, exhibits several advantageous traits such as rapid growth, high salinity tolerance, wide pH adaptability, and efficient resource acquisition and utilization compared to native plant species. The invasion mechanism of SA in China is influenced by both biotic and abiotic factors, with biotic factors, especially its reproductive capacity, considered crucial for its successful invasion. The presence of introduced invasive species in coastal wetlands brings both positive and negative impacts. Hence, it is crucial to consider both the benefits and drawbacks associated with invasive species. The distribution of SA varies across different provinces in temperate and sub-tropical coastal China. Notably, Zhejiang, Jiangsu, Fujian, and Shanghai are the provinces with the largest *Spartina alterniflora* areas, accounting for over 90% of the total *Spartina alternflora* area. SA’s adaptability and competitive traits enable rapid growth and losing crucial microbial, plant, and animal diversity. It displaces native salt marsh plants, encroaches into mangrove gaps, and transforms mudflat habitats. SA invades ecosystems, out-competing native plants like *Phragmites australis*, particularly in areas with varying salinity levels. The invasion negatively affects benthic fauna, leading to a decline in mollusk density and trophic diversity. Soil microbial communities are also altered by SA invasion, with bacterial and fungal composition changes. The impact of SA invasion on carbon storage in coastal wetlands is still uncertain. Some studies suggest that SA enhances carbon storage by promoting organic matter input and altering soil properties, while others argue that it weakens this function. The invasion of SA can lead to changes in carbon–nitrogen ratios, soil microbial environments, and emissions of carbon-containing greenhouse gases. The effects of SA invasion on soil inorganic carbon and carbon dynamics in coastal wetlands require further investigation.

The presence of SA in China has significantly transformed the composition and operation of coastal wetlands. To effectively manage, enhance, or eliminate SA, it is crucial to adopt a comprehensive and adaptive strategy that considers the long-term implications and specific circumstances within each province of China.

## Figures and Tables

**Table 1:** Biotic and Abiotic factors contribute to the spread of *Spartina alterniflora* in China.

Biotic Components	Abiotic Components
Broad Niche	Purposeful introduction
Competitive Nature	Niche Space
Higher Biomass	Apposite Climate
Ability to Reproduce	Suitable Habitat Environment
Rapid spread mechanism	

Data Source: [32,36,37].

**Table 2:** Analysis of soil organic carbon levels in Chinese salt marshes dominated by *Spartina alterniflora*.

Study Area	Invaded Species	Invasion Time	SOC	Reference
Hangzhou Bay Wetland (Zhejiang Province)	*Scirpus mariqueter* and *Phragmites australis*	No data	13.50	[71]
Minjiang River estuary (Fujian Province)	*Cyperus malaccensis*	12 years	14.3–23.5	[66]
Yancheng Natural Reserve (Jiangsu Province)	*Scirpus mariqueter* and *Phragmites australis*	Not Available	4.75	[65]
Jiuduansha Wetland National Nature Reserve (Shanghai Province)	*Scirpus mariqueter*	Not Available	6.77	[64]
Dongtan wetland of Chongming Island (Shanghai Province)	*Scirpus mariqueter* and *Phragmites australis*	12	20.31	[68]
Wanggang estuarine (Jiangsu Province)	*Suaeda salsa*	14	4.90	[69]
Yancheng Natural Reserve (Jiangsu Province)	*Suaeda salsa*	10	13.5	[72]
Xinyang Wetland (Zhejiang Province)	*Suaeda salsa*	12	6.35	[73]

## Data Availability

The datasets used during the study are available from the corresponding author on request.
